# Loxoscelism: Cutaneous and Hematologic Manifestations

**DOI:** 10.1155/2019/4091278

**Published:** 2019-03-20

**Authors:** Ngan Nguyen, Manjari Pandey

**Affiliations:** ^1^Department of Internal Medicine, University of Tennessee Health Science Center, 956 Court Ave., Suite H314, Memphis, TN 38163, USA; ^2^Department of Hematology and Oncology, West Cancer Clinic, 7945 Wolf River Blvd, Germantown, TN 38138, USA; ^3^Department of Hematology and Oncology, University of Tennessee Health Science Center, Memphis, TN 38163, USA

## Abstract

**Background:**

Brown recluse spider (BRS) envenomation can lead to significant morbidity through severe local reaction and systemic illness including acute hemolytic anemia, rhabdomyolysis, disseminated intravascular coagulopathy (DIC), and even death. We aim to describe the clinical features and the roles of antibiotics and steroids in the treatment of loxoscelism.

**Methods:**

We retrospectively identified nine patients (pts) at our institution who were admitted with moderate to severe loxoscelism. A chart review was performed to highlight important clinical features and effect of interventions.

**Results:**

Nine pts (age 18 to 53) presented with fever (6), rash (9), pain/swelling (4), and jaundice (2). Of these, 6 pts had antecedent spider bites documented. Five pts were discharged from Emergency Room (ER) with oral antibiotics for “cellulitis” and were readmitted with severe systemic symptoms, with almost half (45%) of the pts being admitted to the intensive care unit. The most common admission diagnosis was sepsis secondary to cellulitis (6). Four pts developed worsening dermonecrosis, and 3 received prompt incision and drainage (I&D) with debridement. Hemolytic anemia developed around day 5 after spider bite (average); the lowest mean hemoglobin level was 5.8g/dL, with average drop of 3.1 g/dL. Direct antiglobulin test (DAT) (for both complement and surface immunoglobulin) was positive in 4 out of 9 patients. Four pts received glucocorticoid therapy for their hemolytic anemia. The use of steroid and intravenous immunoglobulin (IV Ig) did not seem to show a difference in the time of recovery although those who received steroids required less blood transfusion (2.1 units less). All pts had a complete recovery within two weeks.

**Conclusion:**

Treatment of systemic loxoscelism involves aggressive supportive care including appropriate wound management, blood transfusions, intravenous fluid replacement, and appropriate antibiotic coverage. It is unclear at this time if glucocorticoids or IVIg has any beneficial impact on the treatment of severe loxoscelism.

## 1. Introduction

Spiders of the genus Loxosceles have a common name “brown spiders.” Among these, the brown recluse spiders, or Loxosceles reclusa, have gained notoriety in the medical literature. Their bites can cause clinical manifestations like skin necrosis and occasionally severe systemic manifestations such as acute hemolytic anemia, rhabdomyolysis, and DIC. The brown recluse spider is commonly found in homes in endemic areas, in the United States; this includes parts of South, West, and Central Midwestern United States [1]. They prefer isolated spaces such as closets, attics, or basement. Most of the recluse bites occur only when they feel disturbed or endangered.

Recluse spider venom contains various enzymes which are enriched in phospholipase D, sphingomyelinase, astacin-like metalloproteases, and Inhibitor Cystine Knot peptides [2]. Among these, phospholipase D is unique to Loxosceles and has clinical significance. It exerts its effects by activating complement and inducing neutrophil chemotaxis and apoptosis of keratinocytes. It is known to cause the local effect of dermonecrosis and systemic manifestations including hemolysis, thrombocytopenia, and renal failure [3].

There have been no available commercial tests to detect spider venom [4]. A presumptive diagnosis of spider bites is made based on the history and physical exam findings, while a definitive diagnosis can be considered only if the spider was observed biting and recovered. Otherwise the diagnosis of a Loxosceles spider bite should be considered after ruling out other causes. As a result of usually not being able to apprehend a spider at the time of the bite, the diagnosis can often be missed by clinicians and mislabeled as a skin infection or cellulitis. For patients with systemic findings positive for fever, myalgia, nausea, and/or vomiting, laboratory studies are warranted to look for hemolytic anemia, coagulopathy, and acute kidney injury. To date, there is no definitive intervention or guideline treatment for loxoscelism besides supportive care [5]. In this paper, we retrospectively identified nine patients with moderate to severe loxoscelism treated at our institution. We will focus on both local and systemic manifestations of the disease to highlight the need for high index of suspicion and review the role of commonly used treatments: antibiotics, steroids, and IVIg for loxoscelism.

## 2. Case Reports

### 2.1. Initial Presentations

Nine patients whose ages ranged from 18 to 53 presented with cellulitis. The most common initial presentations were fever (6), rash (9), pain and swelling (4), and jaundice (2) (Table 1). Interestingly, 8 out of 9 patients were female. Six patients could recall an antecedent spider bite and identify the exact location, while the other three had no recollection of a bite. All of them presented with a rash on their distal upper extremities (3), proximal upper extremity (1), or proximal lower extremities (5) (Table 1). Five patients were discharged from the ER with local care and antibiotics, only to be readmitted within a week for worsening systemic symptoms. The most common admission diagnosis was sepsis secondary to cellulitis (6). Four patients were admitted to the intensive care unit (ICU), while five were treated in the regular medicine floor. Patients #2 and 3 developed septic shock and required vasopressor in the ICU. The average hospital stay length was 7.3 days.

### 2.2. General Findings

**Figure 1:**
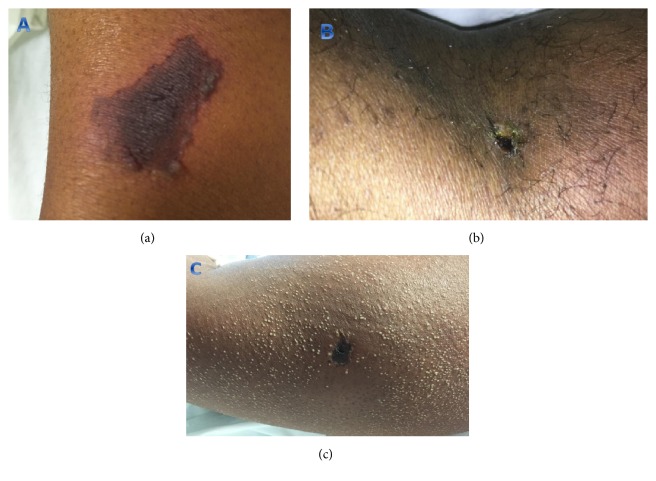
Recluse spider bite with skin necrosis. (a), (b): area of ecchymosis (a) and black eschar (b) after recluse spider bite. (c) Black eschar surrounded by miliary rash as a result of recluse spider bite local reaction.

All patients initially presented with an indurated erythematous rash and received wound care with daily dressing changes during their hospital stay. In four patients, the rash progressed to a local black eschar with surrounding desquamation over an average of 5 days (Figure 1). Three patients with skin necrosis received prompt open incision and drainage and debridement. The average time to recovery after prompt debridement was 3 days. Patient 8 was the only one with skin necrosis who did not receive debridement in her first hospital stay. Thirty days after discharge, her wound in the left inner thigh had progressed into abscess. She had to be readmitted for wound debridement with wound vacuum-assisted closure. The most common bacteria grew in surgical wound culture were Staphylococcus epidermidis (2 out of 4), Pseudomonas (1), and E. coli (1).

Six patients at presentation had fever, tachycardia, and leukocytosis and were diagnosed with sepsis secondary to cellulitis based on systemic inflammatory response syndrome (SIRS) criteria. Patients 2, 3, 4, and 8 had remarkable peak white blood cell count of 50.9, 65, 44.8, and 50.2, respectively. In patients 2 and 7, their sepsis did not improve in spite of broad spectrum antibiotics and aggressive fluid resuscitation and only subsided after surgical debridement was done. In this case series, antibiotic use included vancomycin (55.5%), clindamycin (44.4%), zosyn (22.2%), cefepime (22.2%), doxycycline (22.2%), cephalexin (11.1%), and meropenem (11.1%).

**Figure 2:**
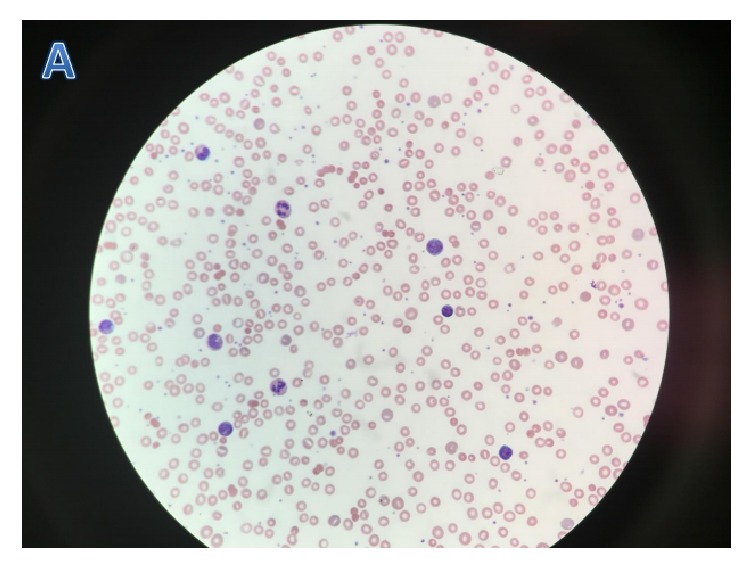
Peripheral blood smears in patients with hemolytic anemia: peripheral smear showed microspherocytes and some increased bands.

Hemolytic anemia occurred in all 9 patients with the lowest mean hemoglobin level of 5.8 g/dL. On average, patients experienced hemolysis beginning day 5 post-bite. Four of them developed hemolysis earlier, on days 2, 3, and 4, while five had hemolysis later, on days 5, 6, and 7. The DAT test was positive for both surface complement component C3 and IgG in 4 out of 9 patients; none of our pts had IgG or C3 alone positivity. Peripheral smears in these patients often showed the presence of microspheres with some increased bands (Figure 2). None of nine patients had palpable hepatosplenomegaly or lymphadenopathy documented on physical exam. Computed tomography (CT) scans of the abdomen and pelvis were not performed. Patients received an average of 3.1 units of pRBC (packed Red Blood Cell) transfusions. Four patients received glucocorticoid therapy for their hemolytic anemia. In addition to steroid, patient 8 received IV Ig therapy.

AKI was present in 3 out of 9 patients (33.3%), and mild transaminitis was found in 5 patients (55.6%) (Table 2). Both AKI and transaminitis resolved at the time of discharge.

There seemed to be no difference in the time to recovery in patients receiving additional steroid or steroid and IV Ig versus those who did not; average time to recovery was within 7 days in all patients. However, those who received steroid were found to require less blood transfusion: 2 vs 3.4 units of pRBCs.

## 3. Discussion

Brown recluse spider bites often occur indoors in uninhabited places such as in basement or closets. The classical dermatologic findings caused by brown recluse spider bites are an initial inflammatory reaction at the bite sites followed by local eschar. Patients who present with a skin lesion centered by two puncture marks and surrounding ecchymosis should be evaluated for possible spider bite [6]. Of note, 8 out of 9 patients in this case series were female. The most common bite location is the proximal lower extremity, then distal upper extremity. No bite was observed on trunk, face, or hands. Location of bite did not seem to affect severity of local reaction or systemic illness. Cellulitis and skin necrosis are the most common findings of local loxoscelism. In this review, 4 out of 9 patients (44.4%) developed skin necrosis. Sphingomyelinase is believed to be the main agent causing skin necrosis as it activates complements and induces apoptosis of keratinocytes [7]. Appropriate wound care, prompt I&D, and debridement are necessary to permit appropriate healing. Of note, patient 7 who developed a black tender eschar continued to spike fevers on three consecutive days in the ICU despite being on broad spectrum antibiotics. Her fever only subsided after debridement was performed. Similarly, patient 8 had a 5x2cm black eschar on her inner thigh at admission, and debridement was deferred. Thirty days after discharge, patient was readmitted due to worsening eschar (now 11x3cm) with abscess formation. She required extensive debridement with wound VAC. These findings suggest that delays in debridement in patients with moderate size of dermonecrosis can lead to progressive skin lesions and delay wound healing. Average time to recovery after prompt debridement in patients with skin necrosis was 3 days. It is also good to note that most necrotic skin lesions are very tender in these patients; thus appropriate pain control is indicated.

Surgical wound cultures showed both Gram positive bacteria such as Staph and Strep and Gram negative rods such as Pseudomonas and E. coli; therefore, antibiotics should have coverage of both Gram positive cocci and Gram negative rods. None of our patients developed bacteremia, DIC, or death as a result of systemic loxoscelism.

Loxoscelism can also cause significant leukocytosis even in the absence of infection; 3 out 9 patients had peak WBC above 40,000/mL (Table 3). While the mechanism of venom-induced leukocytosis is not completely understood, leukocytosis is believed to be partly from the effect of phospholipase D in inducing neutrophil chemotaxis [2]. Further, a high index of suspicion needs to be maintained for development of systemic loxoscelism. Five of the nine patients in our series were discharged from ER with no planned follow-up. Equally important is to consider that patients with systemic findings such as fever, myalgia, and jaundice warrant laboratory studies to assess for hemolytic anemia, rhabdomyolysis, and kidney injury. Acute intravascular hemolysis has been described as the main process of systemic loxoscelism [8]. The pathogenesis of venom-induced hemolysis is not completely understood, but it is believed to be from direct sphingomyelinase venom toxin-induced-hemolysis and complement mediated immune destruction [2]. In our review, hemolysis was present in all 9 patients, accompanied by markers of hemolysis such as elevated LDH, hyperbilirubinemia, and low haptoglobin levels (<8 mg/dL). Not all pts had positive Direct Antiglobulin Test (DAT). In severe forms of loxoscelism, hemoglobin level can become extremely low (< 6 g/dL), as seen in 4 out of 9 patients in our review. On average, each patient with hemolytic anemia required 3.1 units of pRBCs. Further, 4 of 9 patients had positive DAT test for both IgG and complement, (in a previous report some pts had isolated IgG or complement DAT positive [9]) suggesting that autoimmune mechanism and complement mediated-immune destruction both may play a role in the development of intravascular hemolysis. Corticosteroids have been used in patients with moderate to severe hemolysis, but literature supporting the use of steroid in the treatment of hemolysis in loxoscelism is sparse [10]. In this case series, we found no significant difference in the time of recovery from hemolysis in patients with and without steroid use, but those who received steroid were found to require less blood transfusion. Four patients who received IV solumedrol required only 2 units of pRBCs compared to 4.1 units of pRBCs in those who did not receive steroid. While the use of corticosteroid remains unproven in the management of loxoscelism, it may have some benefit and remains a subject requiring further investigation. All patients had full resolution of hemolytic anemia within two weeks, and there was no recurrence of hemolysis for up to two years of follow-up.

AKI was only observed in 3 of 9 patients (33.3%) with an average rise in serum creatinine of 1.03 from baseline. Hemolysis, direct toxin-induced acute tubular necrosis, rhabdomyolysis, and hypoperfusion from sepsis have been postulated as the cause of kidney injury in loxoscelism [11]. In our review, AKI did not correlate with the severity of hemolysis or sepsis and resolved after fluid and steroid administration. Therefore, it is difficult to tell whether steroid had any impact on the treatment of AKI. Electrolyte abnormalities can be seen in loxoscelism; hypocalcemia was noted in 3 out of 9 patients (33.3%). Hypokalemia and hypomagnesemia were present in one patient. Mild transaminitis with AST and ALT levels below 100 was present in 5 patients with hemolysis. Transaminitis in patients with systemic loxoscelism could be from a complication of hemolysis and/or sepsis induced hypoperfusion. Transaminitis resolved as hemolysis and sepsis resolved. While only 3 out of 9 patients had their CPK checked (found to be within the normal limit), we suggest checking CPK levels for all the patients because rhabdomyolysis is a known complication of systemic loxoscelism.

## 4. Conclusion

Diagnosis of systemic loxoscelism is often delayed; improved patient education and close follow-up for systemic manifestations are warranted. Most patients develop the clinical syndrome within 7-10 days of bite; common symptoms include fever, rash, and jaundice and require further laboratory evaluation to look for hemolytic anemia, AKI, rhabdomyolysis, or DIC. The favorable clinical outcomes in our case-series suggest that treatment of systemic loxoscelism is mainly supportive. Appropriate wound care with prompt surgical debridement if skin necrosis develops facilitates rapid wound healing. In regard to the use of antibiotics, the clinician should consider Gram positive cocci and Gram negative rod coverage. While the process appears to be self-limiting, use of steroid may reduce severity and need for transfusions.

## Figures and Tables

**Table 1:** Admission data and patient characteristics.

Admission data and patient characteristics	
	Incidence Rate; % (n)

Age in years (median, range)	30,18-53

Gender	

Male	11.1% (1)

Female	89.9% (8)

Antecedent spider bite documented	

Yes	67.7% (6)

No	33.3% (3)

Bite location	

Distal upper extremity	33.3% (3)

Proximal upper extremity	11.1% (1)

Proximal lower extremity	55.6% (5)

Admission type	

General Med-Surg	55.6% (5)

ICU	45.4% (4)

Hospital stay length in days (mean ± SD)	7.3 ± 2.8

Chief complaints at admission	

Fever	66.7% (6)

Rash	100% (9)

Pain and swelling	44.4% (4)

Jaundice	22.2% (2)

**Table 2:** Clinical findings and associated treatments.

Clinical findings	Percentage (n) of patients
Skin necrosis	44.4% (4)
Sepsis	66.7% (6)
Fever	66.7% (6)
Tachycardia	77.8% (7)
Hypotension	66.7% (6)
Hemolytic anemia	100% (9)
Acute kidney injury	33.3% (3)
Transaminitis	55.6% (5)

**Table 3:** Laboratory and clinical course of patients with systemic loxoscelism.

	Patient 1	Patient 2	Patient 3	Patient 4	Patient 5	Patient 6	Patient 7	Patient 8	Patient 9
Initial WBC count (x10^3^/mL)	5.6	40.7	17.1	44.8	14	11.4	13	9.3	9.3
Peak WBC count (x10^3^/mL)	15.9	50.9	65	44.8	17.3	29.4	16	50.2	13.4
Lowest platelet (x10^3^/mL)	136	365	152	179	136	124	117	144	320
Initial Hgb (g/dL)	10.1	5.3	3.5	11.7	4.7	12	8.8	9.3	14.2
Lowest Hgb (g/dL)	6.3	5.3	3.5	8.2	4.7	4.4	6.0	7.6	4.8
Onset of hemolysis relative to bite (days)	5	3	4	5	3	7	6	1	6
Hgb at discharge (g/dL)	7.5	8.2	8.8	9.1	11.1	6.9	7.8	12	9.1
Peak Reticulocyte count (%)	3.9	10.8	0.6	3.93	1.6	2.6	2.7	10.5	13.2
Haptoglobin (mg/dL)	<8	<8	Not measured	Not measured	Not measured	67	Not measured	158	197
Peak total bilirubin	16.2	5.2	39.2	0.5	4.3	4.3	2.4	21	5.6
LDH at admission (U/L)	1146	953	2337	328	1633	320	261	566	549
DAT (Surface IgG)	negative	negative	positive	negative	negative	positive	negative	positive	positive
DAT (Surface C3)	negative	negative	positive	negative	negative	positive	negative	positive	positive
Hospital admission	Med-Surg	ICU	ICU	ICU	Med-Surg	Med-Surg	ICU	Med-Surg	Med-Surg
Transfusion (pRBC units)	1	3	4	0	7	3	3	2	2
Corticosteroid use	No	No	Yes	Yes	No	No	No	Yes	Yes
IVIg use	No	No	No	No	No	No	No	No	Yes
Incision and drainage	No	Yes	No	No	No	Yes	Yes	Yes	No

Abbreviations: WBC, White Blood Cell; LDH, Lactate Dehydrogenase; DAT, Direct Antiglobulin Test; pRBCs, packed Red Blood Cells.

## Data Availability

The material supporting the conclusion of this review has been included within the article.

## Abbreviations

BRS: Brown recluse spider
pt: Patient
DIC: Disseminated intravascular coagulopathy
ER: Emergency room
I&D: Incision and drainage
DAT: Direct antiglobulin test
IVIg: Immunoglobulin
ICU: Intensive care unit
SIRS: Systemic inflammatory response syndrome
pRBC: Packed red blood cell
AKI: Acute kidney injury
WBC: White blood cell
LDH: Lactate dehydrogenase.
